# Walking adaptability therapy after stroke: study protocol for a randomized controlled trial

**DOI:** 10.1186/s13063-016-1527-6

**Published:** 2016-08-26

**Authors:** Celine Timmermans, Melvyn Roerdink, Marielle W. van Ooijen, Carel G. Meskers, Thomas W. Janssen, Peter J. Beek

**Affiliations:** 1 MOVE Research Institute Amsterdam, Department of Human Movement Sciences, Vrije Universiteit Amsterdam, Van der Boechorststraat 9, Amsterdam, 1081 BT The Netherlands; 2 Amsterdam Rehabilitation Research Center, Reade, Overtoom 283, Amsterdam, 1054 HW The Netherlands; 3 VU Medical Centre, Department of Rehabilitation Medicine, De Boelelaan 1118, Amsterdam, 1081 HZ The Netherlands

**Keywords:** Exercise, Rehabilitation, Stroke, Therapy, Walking adaptability, Walking speed

## Abstract

**Background:**

Walking in everyday life requires the ability to adapt walking to the environment. This adaptability is often impaired after stroke, and this might contribute to the increased fall risk after stroke. To improve safe community ambulation, walking adaptability training might be beneficial after stroke. This study is designed to compare the effects of two interventions for improving walking speed and walking adaptability: treadmill-based C-Mill therapy (therapy with augmented reality) and the overground FALLS program (a conventional therapy program). We hypothesize that C-Mill therapy will result in better outcomes than the FALLS program, owing to its expected greater amount of walking practice.

**Methods:**

This is a single-center parallel group randomized controlled trial with pre-intervention, post-intervention, retention, and follow-up tests. Forty persons after stroke (≥3 months) with deficits in walking or balance will be included. Participants will be randomly allocated to either C-Mill therapy or the overground FALLS program for 5 weeks. Both interventions will incorporate practice of walking adaptability and will be matched in terms of frequency, duration, and therapist attention. Walking speed, as determined by the 10 Meter Walking Test, will be the primary outcome measure. Secondary outcome measures will pertain to walking adaptability (10 Meter Walking Test with context or cognitive dual-task and Interactive Walkway assessments). Furthermore, commonly used clinical measures to determine walking ability (Timed Up-and-Go test), walking independence (Functional Ambulation Category), balance (Berg Balance Scale), and balance confidence (Activities-specific Balance Confidence scale) will be used, as well as a complementary set of walking-related assessments. The amount of walking practice (the number of steps taken per session) will be registered using the treadmill’s inbuilt step counter (C-Mill therapy) and video recordings (FALLS program). This process measure will be compared between the two interventions.

**Discussion:**

This study will assess the effects of treadmill-based C-Mill therapy compared with the overground FALLS program and thereby the relative importance of the amount of walking practice as a key aspect of effective intervention programs directed at improving walking speed and walking adaptability after stroke.

**Trial registration:**

Netherlands Trial Register NTR4030. Registered on 11 June 2013, amendment filed on 17 June 2016.

**Electronic supplementary material:**

The online version of this article (doi:10.1186/s13063-016-1527-6) contains supplementary material, which is available to authorized users.

## Background

The ability to adapt walking to environmental circumstances, such as the ability to avoid obstacles and to secure safe foot placement in a cluttered environment, is a prerequisite for safe walking in everyday life circumstances. This gait adaptability or walking adaptability [1, 2] is often reduced after stroke [3, 4], which might contribute to the high fall risk in this population [5]. There is thus a clear need to improve this aspect of walking ability in people with stroke.

One of the most promising exercise therapies that include practice of walking adaptability is task-specific gait training [5]. Task-specific gait training refers to the practice of associating functional tasks with walking. The benefits of task-specific training in stroke rehabilitation have been demonstrated in several studies [6–8]. Besides task-specific training, context-specific training is a well-accepted rehabilitation principle after stroke, suggesting that training should target the goals relevant for the needs of people with stroke attuned to their environmental circumstances [6, 8]. Hence, including walking adaptability exercises in training interventions aimed at improving safe community ambulation seems appropriate and potentially beneficial for people with stroke.

**Fig. 1:**
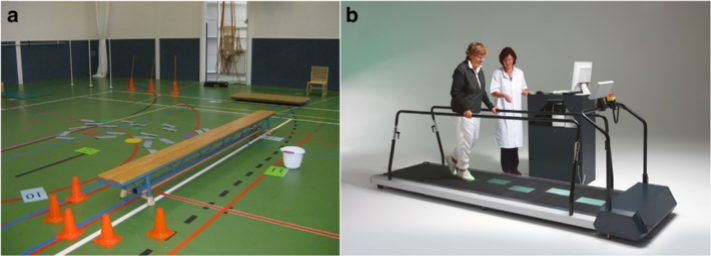
Snapshots of the two interventions aimed at improving walking speed and walking adaptability: (**a**) obstacle course of the overground FALLS program; (**b**) targeted-stepping exercise of treadmill-based C-Mill therapy

The FALLS program [9] is one such task-specific and context-specific type of overground training intervention, which integrates the practice of complex situations of community walking, such as walking over an obstacle course (Fig. 1a). The FALLS program is based on the Nijmegen Falls Prevention Program, which was designed for community-dwelling older adults with a history of falling, and was shown to reduce the number of falls in this population [10, 11]. Although the effectiveness of the FALLS program needs to be determined in people with stroke, it has been shown to be feasible for this population [9].

C-Mill therapy is another promising example of task-specific and context-specific training with an emphasis on walking adaptability exercises. The C-Mill (Fig. 1b) is an instrumented treadmill augmented with task-relevant visual context (e.g., obstacles, stepping targets) projected on the treadmill’s surface [12]. This context can be administered in a gait-dependent manner, owing to online monitoring of timing and location of foot placements [13]. The projected obstacles and stepping targets make C-Mill therapy well suited for task-specific and context-specific training because step adjustments are required to adapt to the projected context similar to the step adjustments required to adapt to environmental circumstances during community ambulation. A recent proof-of-concept study showed that C-Mill therapy in the chronic stage after stroke is not only well received by this population, but also beneficial [14]. C-Mill therapy resulted in training-related increments in walking speed and improvements in various other walking-related clinical scores. In addition, the ability to make step adjustments improved (i.e., higher obstacle-avoidance success rates) after 5–6 weeks of C-Mill therapy, and these adjustments required less attention (i.e., reduced dual-task interference), suggesting that the step adjustments evolved in a more automatized manner after a period of C-Mill therapy [15].

Besides task-specific and context-specific training, other key ingredients for effective rehabilitation include variability in practice, feedback of performance, and amount of movement practice [6–8, 16, 17]. Both interventions comprise variability in practice, given their wide variety of tasks and exercises. Moreover, both interventions allow for performance feedback, either by group discussions and direct feedback provided by therapists (FALLS program) or by direct feedback of walking adaptability exercise performance, e.g., visual feedback with regard to obstacle hits (C-Mill therapy). However, treadmill-based C-Mill therapy probably allows for a greater amount of walking practice (defined as the number of steps taken per session), because it incorporates treadmill walking, which has been suggested to elicit more steps per session than overground training [18–22]. In this study, we will empirically test this suggestion, using the amount of walking practice as a process measure.

The study’s aim is to compare the effects of two promising interventions for improving walking speed, walking adaptability, and commonly used clinical measures of walking and balance in persons after stroke: treadmill-based C-Mill therapy [14, 15] and the overground FALLS program [9]. We expect that C-Mill therapy will result in better outcomes than the FALLS program because of the expected greater amount of walking practice per session of equal duration.

## Methods

### Participants

In total, 40 persons who had a stroke will be recruited from the inpatient and outpatient population of rehabilitation center Reade (Amsterdam, The Netherlands) to participate in this study. Inclusion criteria are first-ever stroke ≥3 months ago, walking or balance deficits confirmed by a physician, clinical diagnosis of hemiparesis, age ≥18 years, general walking ability as indicated by a Functional Ambulation Category score ≥3 [23], and the ability to understand and execute simple instructions. Exclusion criteria are orthopedic and other neurological disorders that affect walking (e.g., Parkinson’s disease), other treatments that could influence the effects of the interventions (e.g., recent Botulin toxin treatment of the lower extremity), contra-indication to physical activity (e.g., heart failure, severe osteoporosis), moderate or severe cognitive impairments as indicated by a Mini-Mental State Examination [24] score below 21, or severe uncorrected visual deficits. Persons with stroke who are eligible for participation will be informed about the study by their rehabilitation specialist, both orally and in writing. All participants will provide a written informed consent.

### Study design

**Fig. 2:**
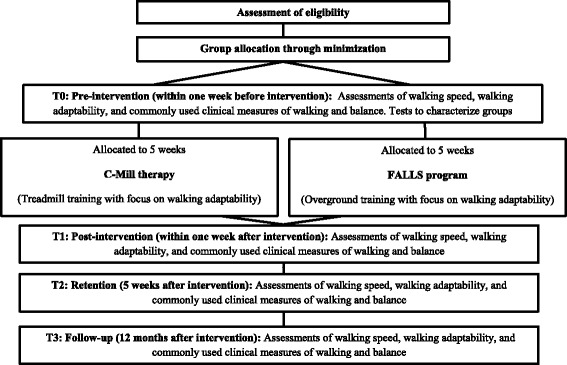
Flow chart of the procedures that participants will undergo

The proposed study is a single-center, parallel group randomized controlled trial with pre-intervention, post-intervention, retention, and follow-up tests to determine the relative efficacy of the interventions: treadmill-based C-Mill therapy and the overground FALLS program. After giving informed consent, participants will be randomly assigned to one of the two interventions using an automated, custom-made minimization algorithm written in MATLAB. The minimization procedure is based on time after stroke, age and Functional Ambulation Category score to balance groups for these stratification factors. The research assistant will enter the data for randomization in the algorithm and the participant will subsequently be informed about the resulting group allocation before the pre-intervention tests. Subsequently, the assessor will schedule the participants for the assigned 5 week intervention program. Pre-intervention tests (T0) to characterize groups and obtain baseline values of primary and secondary outcome measures will be performed one week prior to the intervention program. Within one week after completing the intervention, post-intervention tests (T1) will be performed. The same tests will be conducted 5 weeks (retention tests, T2) and 12 months (follow-up tests, T3) after completing the intervention. All assessments will be performed at the rehabilitation center. Because of the nature of the intervention studied, therapists and participants cannot be blinded to group allocation. The assessor will also not be blinded to group allocation, because of pragmatic constraints related to the planning of assessments and therapy sessions. Figure 2 shows a flow chart of the procedures that participants will undergo at T0, T1, T2 and T3.

### Interventions: treadmill-based C-Mill therapy and the overground FALLS program

**Fig. 3:**
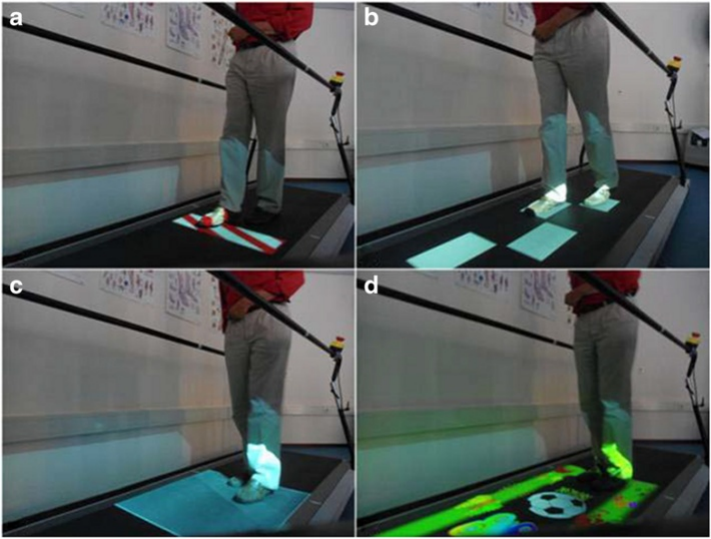
Exercises of treadmill-based C-Mill therapy: (**a**) obstacle avoidance; (**b**) visually guided stepping to a sequence of stepping targets; (**c**) acceleration and deceleration evoked by a moving walking area; (**d**) functional and interactive walking adaptability game (adopted from Van Ooijen et al. [20])

C-Mill therapy is a structured treadmill training program with a specific emphasis on practicing walking adaptability (as detailed in Table 1 and Additional file 1), using gait-dependent augmented-reality content projected on the instrumented treadmill surface to elicit step adjustments [1, 13–15, 25–28]. Figure 3 shows various exercises of C-Mill therapy, including exercises to practice avoidance of projected visual obstacles (Fig. 3a), exercises to practice accurate foot placement on a step-to-step basis by walking to a regular or irregular sequence of visual stepping targets (Fig. 3b), exercises to practice acceleration and deceleration by maintaining position within a projected walking area that moves along the treadmill (Fig. 3c), and a functional and interactive walking adaptability game (Fig. 3d). C-Mill therapy is a patient-tailored type of training in that the therapist can adjust the difficulty of the different exercises by manipulating content parameters as the obstacle size and available response time for obstacle negotiation, the variation in the sequence of stepping targets, and the degree of acceleration and deceleration of the moving walking area. As progressive training has previously been shown to have beneficial effects [29–31], therapists are instructed to increase the difficulty of C-Mill exercises as tolerated by the participant, by either changing content parameters or increasing the belt speed, as described in the pre-defined training protocol (Table 1). To assist therapists in progressively scaling the C-Mill therapy sessions, the participant’s perceived fear and difficulty levels during the sessions will be assessed at a scale from 0 (no fear or not difficult) to 10 (much fear or very difficult), as well as their rating of perceived exertion using the Borg scale (range 6–20, [32]). Furthermore, the pre-defined protocol will guide therapists to vary C-Mill exercises, both in terms of content and the order in which the exercises will be performed, inspired by recent insights into motor learning showing superior transfer and retention effects with variability in practice [16]. C-Mill therapy will be performed in groups of two persons with stroke supervised by one therapist. Therapy sessions will last 1.5 hours each, divided in exercise blocks of 3–8 min, during which the participants alternately train and rest (Table 1).

**Table 1:** Pre-defined protocol for treadmill-based C-Mill therapy

Setting	Groups of two participants for 90 min; participants will alternately train and rest.
Frequency	Twice weekly treadmill training program with specific emphasis on walking adaptability.
Therapy	In the first week, a combination of obstacle avoidance (avoiding visual obstacles projected on the treadmill), practice of accurate foot placement on a step-to-step basis (walking to a regular or irregular sequence of visual stepping targets), and a functional and interactive walking adaptability game (game with the theme ‘beach’ or ‘forest’) will be performed. In weeks 2–5, the combination of obstacle avoidance, accurate foot placement on a step-to-step basis and the functional and interactive walking adaptability game will be complemented by walking speed adaptations (acceleration and deceleration evoked by a moving walking area).
	Participants will start in week 1 at a comfortable walking speed; this speed will be gradually increased during the 5 week period. The weekly increase of the walking speed will be 10 %, provided that the therapy remains safe and is tolerated by the participant. Besides the walking speed, the difficulty of C-Mill exercises will be gradually increased, as tolerated by the participant.
Therapist	C-Mill therapy will be provided by a single therapist, an expert in C-Mill therapy. The therapists involved in the C-Mill therapy were all trained with regard to operating the C-Mill and to the specific guidelines of the intervention before the study started. Most therapists were already experienced C-Mill users before the study started. The therapists regularly meet the research assistant to ensure adherence to the protocol (Additional file 1).

**Fig. 4:**
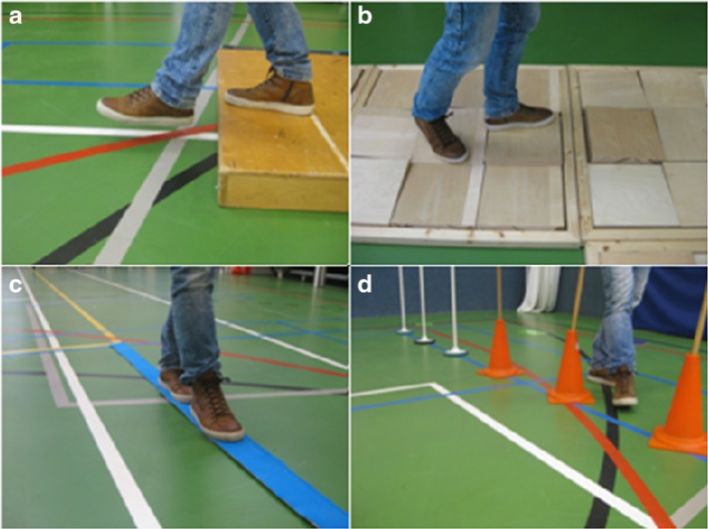
Exercises of the obstacle course of the overground FALLS program: (**a**) obstacle avoidance; (**b**) walking over uneven terrain; (**c**) tandem walking; (**d**) slalom walking

The FALLS program [9] is an overground therapy program aimed at reducing the number of falls in people with stroke by practicing walking adaptability, among other aspects (as detailed in Table 2 and Additional file 2). Figure 4 shows various exercises of this pre-defined FALLS program, including exercises to practice obstacle avoidance (Fig. 4a), exercises to practice foot placement while walking over uneven terrain (Fig. 4b), tandem walking (Fig. 4c), and slalom walking (Fig. 4d). These exercises must also be performed while cognitive and motor dual-tasks are imposed, as well as under visual constraints. In addition, the program incorporates exercises to simulate walking in a crowded environment and to practice falling techniques (one session per week). The FALLS program was originally performed in groups of six persons with stroke, with two or three therapists per group in therapy sessions lasting 2 hours each [9]. Following design considerations for this study (as detailed in the next section), the FALLS program will be performed in groups of four to six persons with two or three therapists per group, with sessions lasting 1.5 hours, including rest.

**Table 2:** Pre-defined protocol for the overground FALLS program

Setting	Groups of 4–6 participants for 90 min, participants will alternately train and rest.
Frequency	Twice weekly overground training program, which incorporates walking adaptability exercises.
Therapy	The first therapy session of the week will be devoted to an obstacle course that simulates potential challenging situations of daily life. The obstacle course facilitates practicing balance, gait, and coordination, and mimics activities of daily life with high fall risk, such as walking over obstacles, uneven terrain, slalom walking and tandem walking. The obstacle course will also be negotiated while imposing cognitive and motor dual-tasks, as well as under visual constraints.
	The second therapy session of the week will include walking exercises and practice of fall techniques. The walking exercises simulate walking in a crowded environment. Adjustments in walking speed and direction are required during these exercises and collisions with other people challenge balance. The practice of fall techniques is based on martial arts techniques and will include falling forwards, backwards, and laterally. The level of difficulty will be gradually enhanced by increasing fall height (from sitting on a safety mat to stance height).
Therapist	The therapy sessions will be provided by two or three therapists, depending on the size of the group. At least one of therapists is trained in the background, methods, and techniques of the FALLS program. All therapists involved in the FALLS program are trained and experienced with regard to the program protocol and instructed to follow the specific guidelines of the intervention for the purpose of this trial. The therapists regularly meet the research assistant to ensure adherence to the protocol. (Additional file 2).

Both interventions are matched for therapy duration (90 min), frequency (twice weekly) and therapist attention (mean participant-to-therapist ratio, 2:1). The amount of walking practice per session (defined as the number of steps performed during therapy sessions) will be compared between the two interventions and treated as the process measure. Therefore, the number of steps taken during C-Mill therapy sessions will be registered using the treadmill’s inbuilt step counter, while an observer will count the number of steps taken during the FALLS program offline in a random selection of FALLS program sessions using video recordings of the sessions in question.

Finally, after completing the last session of the intervention, participants will be asked to fill in a purpose-designed questionnaire to register perceived discomfort during and after therapy sessions, as well as their experience with the therapy, to compare the feasibility of the interventions from a participant’s perspective.

### Outcome measures

After group allocation, pre-intervention tests will be performed to assess the baseline values of primary and secondary outcome measures and to collect participant characteristics (sex, age, height, body mass, medication use, co-morbidities, side and location of the lesion, current living situation, daily functioning and the use of assistive devices). The primary outcome measure in this study will be walking speed. Walking speed will be assessed using the 10 Meter Walking Test [33], which has been shown to be a reliable and robust means for measuring walking speed [34].

**Fig. 5:**
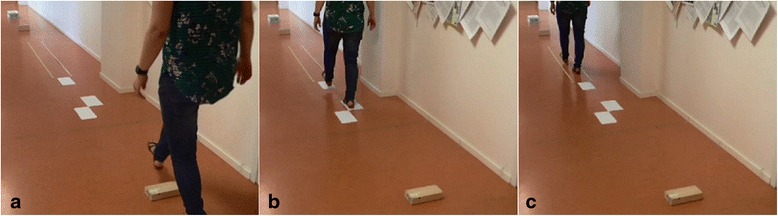
Walking adaptability assessment using the 10 Meter Walking Test with context: (**a**) obstacle avoidance; (**b**) targeted stepping and; (**c**) tandem walking

**Fig. 6:**
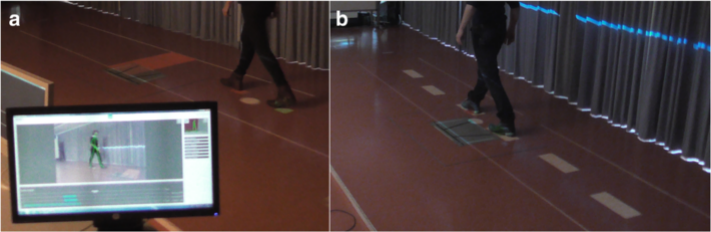
Walking adaptability assessments using the Interactive Walkway: (**a**) avoidance of suddenly appearing obstacles and (**b**) walking to a sequence of stepping targets, both presented on the walking surface in a gait-dependent manner

The secondary outcome measures are inspired by the targeted-stepping and obstacle-avoidance results of Hollands et al. [35] and Van Ooijen et al. [15], underscoring the importance of task-specificity and context-specificity in walking adaptability assessments. Van Ooijen et al. [15] showed enhanced obstacle-avoidance success rates at lower attentional costs after a period of C-Mill walking adaptability therapy, while Hollands et al. [35] showed that measures of targeted stepping were clinically meaningful components in the recovery of functional mobility after stroke. Therefore, the 10 Meter Walking Test will also be performed in combination with context (10 Meter Walking Test with three obstacles, a tandem walking path and three stepping targets) (Fig. 5), a cognitive dual-task (10 Meter Walking Test while counting backwards in steps of three [36]) and both context and dual-task (10 Meter Walking Test with three obstacles, a tandem walking path, and three stepping targets, and while counting backwards in steps of three). Walking adaptability will also be assessed using the Interactive Walkway (Technology4Science, Vrije Universiteit Amsterdam, The Netherlands), a walkway instrumented with multiple Microsoft Kinect for Windows sensors and a projector to present visual context, such as obstacles and stepping targets in a gait-dependent manner (Fig. 6). The walking adaptability evaluation with the Interactive Walkway includes targeted-stepping assessments, obstacle-avoidance assessments, and obstacle-avoidance assessments while counting backwards in steps of three. The obstacle-avoidance assessment of the Interactive Walkway differs from the 10 Meter Walking Test with context in that the Interactive Walkway obstacles can be suddenly presented in a gait-dependent manner, that is, the obstacle suddenly appears at the location where the participant would place his or her foot without adjusting gait. Hence, a step adjustment is always required to avoid the obstacle successfully. Moreover, this step adjustment needs to be performed under high time-pressure demands, which is especially difficult for persons after stroke [37]. The difference between the stepping targets within the 10 Meter Walking Test with context and the Interactive Walkway targeted-stepping assessment is that the Interactive Walkway targets are presented in regular and irregular sequences of visual stepping targets based on participants’ self-selected step length. In this way, it is possible to evaluate foot placement errors on a step-to-step basis for each participant. The 10 Meter Walking Test scores and Interactive Walkway assessment scores will be given in seconds required to complete each test, as well as in the number of errors made during the obstacle crossings, targeted stepping, and tandem walking. The cognitive dual-task, a serial-3 subtraction task, will be analyzed by counting the number of subtractions, as well as the number of mistakes made (dual-task performance [DTP]). Subtraction-task performance while walking will be normalized to subtraction-task performance while sitting (i.e., single-task control condition). These walking adaptability evaluation tools are expected to be sensitive and specific for finding improvements after walking adaptability interventions.

Secondary outcome measures are drawn from a comprehensive set of common clinical measures to determine walking ability, balance, and other walking-related constructs, including Timed Up-and-Go test [38] and Functional Ambulation Category [23]. The obstacle-avoidance subtask of the modified Emory Functional Ambulation Profile will be performed [39], a conventional clinical test closely related to the construct of walking adaptability. The modified Emory Functional Ambulation Profile is reliable and valid for use in people with stroke [40]. Balance will be assessed using the Berg Balance Scale, which provides a psychometrically sound measure of balance impairment for use in post-stroke assessment [40, 41]. Executive function will be assessed using the valid and reliable Trail Making Test [42]. Balance confidence will be assessed with the Activities-specific Balance Confidence scale, a questionnaire measuring balance confidence in performing specific activities, which has good test-retest reliability and validity [43, 44]. Self-reported limitations in walking will be assessed using the Walking Questionnaire [45], which targets experienced limitations in indoor and outdoor walking relative to pre-stroke walking limitations. Finally, the Nottingham Extended Activities of Daily Living scale [46–48] will be used to assess activities of daily living. Table 3 provides an overview of the tests that will be performed at T0, T1, T2 and T3.

**Table 3:** Overview of all tests performed at T0, T1, T2 and T3

Primary outcome measure
10 Meter Walking Test (m/s)
Secondary outcome measures
10 Meter Walking Test with context (m/s, number of errors)
10 Meter Walking Test with a cognitive dual-task (m/s, DTP)
10 Meter Walking Test with context and a cognitive dual-task (m/s, number of errors, DTP)
Interactive Walkway targeted-stepping assessment (m/s, number of errors)
Interactive Walkway obstacle-avoidance assessment (m/s, number of errors)
Interactive Walkway obstacle-avoidance assessment with a cognitive dual-task (m/s, number of errors, DTP)
Timed Up-and-Go test (m/s)
Functional Ambulation Category (3–5)
Obstacle-avoidance subtask of the modified Emory Functional Ambulation Profile (m/s)
Berg Balance Scale (0–56)
Activities-specific Balance Confidence scale (0–100 %)
Trail Making Test (s)
Walking Questionnaire
Nottingham Extended Activities of Daily Living scale (0–66)

Finally, the number of steps taken per therapy session will be recorded, since we expect that the amount of walking practice per session (defined as the number of steps performed during therapy sessions) will be higher for treadmill-based C-Mill therapy than for the overground FALLS program. This expectation will be tested by comparing this process measure between the two intervention groups.

### Sample size

The primary outcome measure in this study will be walking speed. Previous clinical trials in people with stroke by Yang et al. [49] and Jaffe et al. [50] showed greater improvements in walking speed after treadmill training in a complex and challenging virtual reality environment than after, respectively, conventional treadmill training and overground obstacle-avoidance training [49, 50]. Unfortunately, effect sizes and required sample sizes for a controlled clinical trial with multiple comparisons cannot be estimated from the results of these studies, but both reported significant between-group differences in walking speed with small sample sizes of 9 to 10 participants in each intervention group. The study of Yang et al. [49] allows for a sample size calculation for post-hoc analyses for significant group effects on walking speed with independent *t* tests. Based on those results, we aim for a relative, clinically relevant, improvement in walking speed of 0.50 km/h (*∆*) with a common standard deviation (*SD*) of 0.47 km/h, which results in a sample size of 14 participants in each group to achieve 80 % power with a two-tailed *α* of 0.05, i.e., following

$$ N=\frac{2S{D}^2{\left({Z}_{\alpha }+{Z}_{\beta}\right)}^2}{\varDelta^2} $$

[51]. Considering a drop out of 10–25 %, we chose to increase our sample to 20 participants in each intervention group to be on the safe side for establishing the relative efficacy of the two interventions in terms of improvements in walking speed.

### Data analysis

Descriptive group statistics will be used to characterize the two intervention groups in terms of sex, age, height, body mass, Mini-Mental State Examination, Functional Ambulation Category, medication use, co-morbidities, side and location of the lesion, current living situation, daily functioning and the use of assistive devices, as well as perceived discomforts during and after therapy sessions and participant’s experience with the therapy. An independent *t* test will be used to compare the mean number of steps taken per session between the two interventions.

Primary and secondary longitudinal outcome measures that are normally distributed will be analyzed using repeated-measures ANOVA with the between-subject factor group (two levels: C-Mill therapy and the FALLS program) and the within-subject factor time (four levels: pre-intervention [T0], post-intervention [T1], retention [T2], and follow-up [T3] tests). Post-hoc analysis using independent *t* tests between groups per time level will be performed in case of significant interaction effects. For ordinal or non-normal distributed variables, we will use Mann–Whitney *U* tests and Friedman tests to evaluate possible main effects of group and time, respectively. To analyze possible interactions between groups and times, we will apply Kruskal–Wallis tests to change scores (i.e., relative to the previous time level) at T1, T2, and T3. When significant, Mann–Whitney *U* post-hoc tests will be performed to identify between-group differences in change scores per time level. Significant effects are assumed for *P* < 0.05. Data will be analyzed as randomized. Missing data will be imputed using the data from the last available measurement.

## Discussion

This randomized controlled trial will evaluate the relative effects of treadmill-based C-Mill therapy and the overground FALLS program on walking speed and walking adaptability in people with stroke. Although both C-Mill therapy and the FALLS program incorporate practice of walking adaptability and thereby aim at improving community ambulation, and first results are encouraging in this regard [9, 14, 15], it is hypothesized that C-Mill therapy will result in better outcomes than the FALLS program, as a result of the expected greater amount of walking practice owing to treadmill training [18, 20–22]. The results of the study of Moore *et al.* [19] indeed showed significant gains in daily stepping and walking efficacy after treadmill training, compared with conventional physical therapy, which appears to be related to the number of steps taken per session. In this study, we will explicitly test the anticipated greater amount of walking practice with treadmill training by comparing the registered number of steps taken per session between the two intervention groups.

The expected superior outcome of C-Mill therapy relative to the FALLS program may be further mediated by the possibility of tailoring the training to the patient’s needs and progress. During C-Mill therapy, the therapist can adjust the difficulty of the different exercises by manipulating content parameters, such as the variation in the sequence of stepping targets, the obstacle size and available response time for obstacle negotiation, and the degree of acceleration and deceleration of the moving walking area. As progressive training has superior effects [29–31], this patient-tailored challenge of C-Mill therapy might be beneficial, compared with the FALLS program. Conversely, the use of real obstacles and context and the practice of falling techniques might favor outcomes of the FALLS program compared with C-Mill therapy for its superior context-specificity.

A methodological strength of this study is that both interventions will be matched for therapy duration, frequency, and therapist attention. This means that if there is a superior effect on walking adaptability and walking speed of one of the interventions, this will be realized by the same investment in time and resources. Furthermore, both interventions implicitly utilize and train the direct visuolocomotor control of walking in an enriched environmental context [52, 53], allowing for a direct and natural visuolocomotor control in which the point of gaze is typically coupled to future foot placement locations. The two interventions in this study are similar with regard to visuolocomotor control of step adjustments relative to environmental context (e.g., real obstacles in the FALLS program, real visual obstacles in C-Mill therapy). The proposed trial of Hollands *et al.* [54] also testifies to the growing interest in the use of visual cues for task-specific gait training, thereby also implicitly training visuolocomotor control [54]. Hollands *et al.* intend to compare usual care without visual cues to overground visual cue training and treadmill visual cue training (using the C-Mill) in persons with stroke to examine the feasibility of task-specific locomotor practice incorporating visual cues. Therefore, our study, in combination with the study of Hollands *et al.* [54], might underpin the importance of visuolocomotor control in gait rehabilitation, as well as the potential surplus value of a treadmill in that regard.

A limitation of this study is that it involves only one center. This might influence the generalizability of the research results to other rehabilitation centers. Another limitation of this study is the non-blinding of the assessors. To reduce potential influence of this limitation on the outcomes, instructions will be standardized and tasks will be computerized when possible.

In summary, this study will shed light on the effects of treadmill-based C-Mill therapy compared with the overground FALLS program and thereby on the relative importance of the amount of walking practice as an important ingredient of effective interventions of walking speed and walking adaptability after stroke. Hence, the results of this study will be important in optimizing effective intervention programs directed at improving walking speed and walking adaptability after stroke.

### Trial status

Recruitment commenced in 2013 and is ongoing. Results of this study are expected in 2017.

## Abbreviation

ANOVA, analysis of variance; DTP, dual-task performance

## Additional files

Additional file 1: C-Mill therapy treatment booklet for therapists. (PDF 166 kb) Additional file 2: FALLS program treatment booklet for therapists. (PDF 182 kb)
