# Supplementary material for: Walking adaptability therapy after stroke: study protocol for a randomized controlled trial
Source: Trials. 2016 Aug 26;17(1):425. doi: 10.1186/s13063-016-1527-6 (PMC5002097; doi:10.1186/s13063-016-1527-6)
Supplement: Additional file 1: — C-Mill therapy treatment booklet for therapists. (PDF 166 kb) [file 13063_2016_1527_MOESM1_ESM.pdf]

Additional file 1: C-Mill therapy treatment booklet for therapists

**Training 1**<br>Name:__________________	Date:__________	
**0: Warming up**<br>- 5 min (max) walking without visual context projected on the belt's surface<br><br>-Determine comfortable walking speed	**Duration:**<br>______________min<br><br>**Comfortable walking speed**<br><br>______________km/h	
**REST**: 5 min (minimum)<br>The participant sits on a chair while the other participant is training on the C-Mill.<br><br>The therapist fills in the questions.	**Duration:**<br>____________________________________min<br>**RPE-score:**<br>_________________________________<br>At the end of the resting period	
**1: Walking to a sequence of visual stepping targets with random variation**<br><br>- 7 min (max)<br>- Follow one's own stepping pattern with random variation ('Hit the white stepping targets').<br>- 3 entry levels, vary the percentage random variation and choose the percentage that is challenging but doable for the participant.<br>- level 1: 20% variation<br>- level 2: 30% variation<br>- level 3: 40% variation<br><br>- At comfortable walking speed	**Duration**:<br>______________________________min<br><br>**Variation in the stepping pattern /entry level**:____________%<br><br>**How scary was it for the participant (1-10)?** _____________<br>**1 = not at all scary, and 10 = very scary**<br>**How difficult was it for the participant (1-10)?** _____________<br>**1 = very easy, 10 = very difficult**<br>**Mark difficulty by therapist (1-10):**<br>_____________	
	**Number of steps**<br>____________	**Distance**<br>____________m
**REST**: 7 min (minimum)<br>The participant sits on a chair while the other participant is training on the C-Mill.<br><br>The therapist fills in the questions.	**Duration:**<br>________________________________min<br>**RPE-score:**<br>_________________________________<br>At the end of the resting period	

**2: Obstacle avoidance**<br><br>- 7 min (max)<br>- Avoiding obstacles ('Avoid the red obstacles')<br>- Obstacle size: 30 cm<br>- Available Response Time (ART) : 1,2,3 and 4 steps in advance, with a lot of variation and all at least ones<br>- Random for LEFT and RIGHT foot<br><br>- At comfortable walking speed	**Duration**:<br>_________________________min<br><br>**Which ART was given the most?**<br>_______________steps<br>**How scary was it for the participant (1-10)? _____________**<br>**1 = not at all scary, and 10 = very scary**<br>**How difficult was it for the participant (1-10)? _____________**<br>**1 = very easy, 10 = very difficult**<br>**Mark difficulty by therapist (1-10):**<br>_____________<br><br>**Number of steps** ____________ | **Distance** ____________m
**REST**: 7 min (minimum)<br>The participant sits on a chair while the other participant is training on the C-Mill.<br><br>The therapist fills in the questions.	**Duration:**<br>_________________________________min<br>**RPE-score:**<br>__________________________________<br>At the end of the resting period
**3: Walking to a sequence of narrow visual stepping targets**<br><br>- 7 min (max)<br>- Follow narrow stepping pattern ('Hit the white stepping targets').<br>- Project one's own stepping pattern on the treadmill and make the step width as narrow as possible. This exercise should be challenging but doable. (When the step length is too small and stepping targets are presented on top of each other, first adjust the step length so that the individual stepping targets will still be visible)<br>- Guideline: we strive to project a pattern with a step width of 0 in training 5. To reach this the projected step width during this training should be 75% of the participants own step width.<br><br>- At comfortable walking speed	**Duration**:<br>______________________min<br><br>**Step width own pattern:**<br>______________________cm<br>**Step width narrow pattern:**<br>______________________cm<br><br>**How scary was it for the participant (1-10)? _____________**<br>**1 = not at all scary, and 10 = very scary**<br>**How difficult was it for the participant (1-10)? _____________**<br>**1 = very easy, 10 = very difficult**<br>**Mark difficulty by therapist (1-10):**<br>_____________<br><br>**Number of steps** ____________ | **Distance** ____________m
**REST**: 7 min (minimum)<br>The participant sits on a chair while the other participant is training on the C-Mill.<br><br>The therapist fills in the questions.	**Duration:**<br>________________________________min<br>**RPE-score:**<br>__________________________________<br>At the end of the resting period

**4: Walking adaptability game**<br><br>- 4 min (max)<br>- Choose one of the games or obstacle courses and set a challenging but doable level.<br>- After the game, bring down the speed slowly<br><br>- At comfortable walking speed	**Duration**:<br>___________________________min<br><br>**Level of game/obstacle course:**<br>easy/medium/difficult<br><br>**How scary was it for the participant (1-10)? _____________**<br>**1 = not at all scary, and 10 = very scary**<br>**How difficult was it for the participant (1-10)? _____________**<br>**1 = very easy, 10 = very difficult**<br>**Mark difficulty by therapist (1-10):**<br>_____________	
	**Number of steps**<br>____________	**Distance**<br>____________m
**END**<br><br>The therapist fills in the questions.	**RPE-score:**<br>_________________________________<br>At the end of the training	

Describe any adverse events during the training session (e.g. Fall):<br>____<br>____<br><br>Remarks:<br>____<br>____
**SAVE TRAINING SESSION**<br>Press ‘SAVE’
