# Supplementary material for: Walking adaptability therapy after stroke: study protocol for a randomized controlled trial
Source: Trials. 2016 Aug 26;17(1):425. doi: 10.1186/s13063-016-1527-6 (PMC5002097; doi:10.1186/s13063-016-1527-6)
Supplement: Additional file 2: — FALLS program treatment booklet for therapists. (PDF 182 kb) [file 13063_2016_1527_MOESM2_ESM.pdf]

Additional file 2: FALLS program treatment booklet for therapists

Training 1: Obstacle Course

Preparation and supplies:
- The obstacle course is arranged according to the plan (see Below)
- 2 stopwatches

The goals for participants:
- Have practiced the obstacle course.
- Can recognize the various parts of the obstacle course in their daily lives.
- Have practiced safe transfers
- Can perform a safe transfer in their daily lives

10 minutes, introduction:
- Introduce therapists
- Introduce participants
- Preview of the therapy
- Discuss general learning objectives

15 minutes, first part of the obstacle course (1-8):
- The first part of the obstacle is demonstrated.
- The correct performance is defined.
- Each participant performs the first part under supervision.

10 minutes, second part of the obstacle course (9-12):
- The second part of the obstacle course is demonstrated.
- The correct performance is defined.
- Each participant performs the second part under supervision.

15 minutes, complete obstacle course (1-12):
- Each participant performs x times the entire course under supervision.

10 minute break

20 minutes, measure baseline score:
- Each participant performs the complete obstacles course and the time needed and number of errors is determined.

10 min, closing:

- Together with the participants the first training will be evaluated.
- Preview to next training

Reade
revalidatie | reumatologie

C-STROKE

Name:_______________________ **Date:** ______________________

	Mark the items that are performed during the training.			
	Part 1 Obstacle course	Part 2 Obstacle course	Complete obstacle course	*Remarks*
1. Irregular sidewalk				
2. Inclined slope				
3. Balance line				
4. Smooth surface				
5. Clothesline				
6. Deep pile carpet				
7. Vase				
8. Artificial turf with bumps				
9. Slope on and off				
10. Cones at the end of Swedish bank				
11. Grasp ball				
12. Over Swedish bank				
13. Small and large stepping targets				
14. Kneeling and putting the ball in the box				
15. Slalom				
16. Tilting board				
17. Reaching for kitchen cabinets				
18. Pirouette				
19. Sitting and standing from low chair				

Describe any adverse events during the training session (e.g. Fall):

____

Remarks:

____

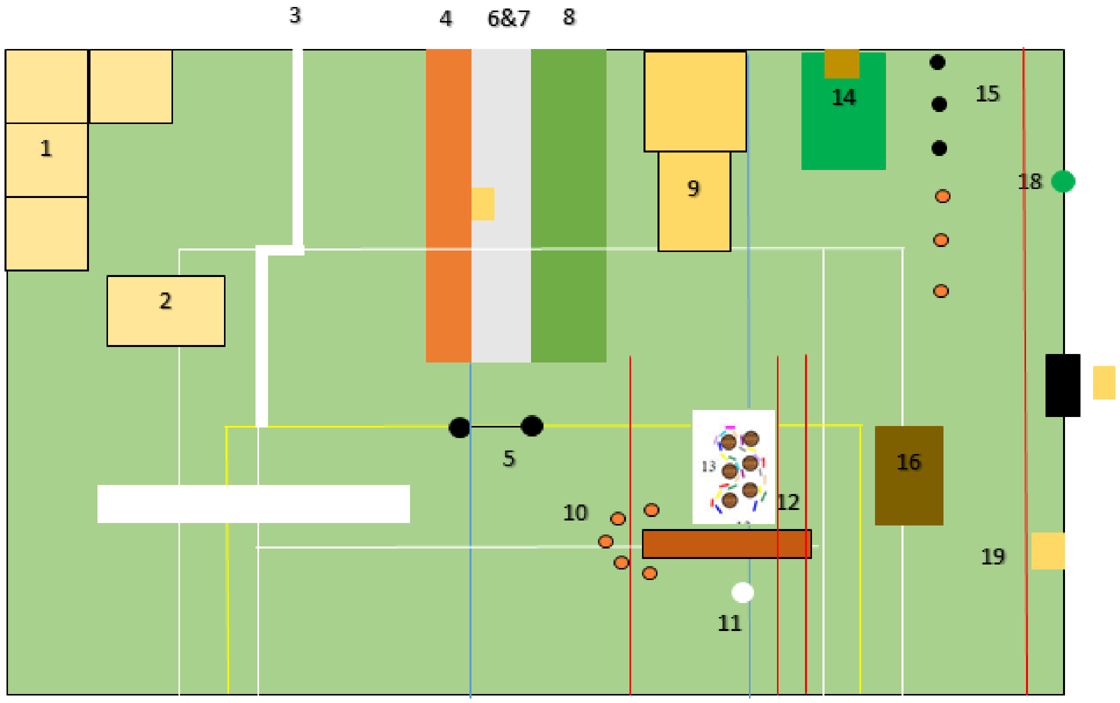

The plan of the obstacle course of the FALLS program. The numbers correspond to the numbers of the exercises in the Table above.

This implementation of the FALLS program is based on the study of Weerdesteyn et al. (2006; 2009) and Van Duijnhoven et al. (2012) [9-11].
